# Modelling spatiotemporal variation in under-five malaria risk in Ghana in 2016–2021

**DOI:** 10.1186/s12936-024-04918-x

**Published:** 2024-04-09

**Authors:** Justice Moses K. Aheto, Lynette J. Menezes, Wisdom Takramah, Liwang Cui

**Affiliations:** 1 Department of Biostatistics, School of Public Health, College of Health Sciences, University of Ghana, Accra, Ghana; 2 WorldPop, School of Geography and Environmental Science, University of Southampton, Southampton, SO17 1BJ UK; 3 College of Public Health, University of South Florida, Tampa, USA; 4 The West Africa Mathematical Modeling Capacity Development (WAMCAD) Consortium, Accra, Ghana; 5 Department of Internal Medicine, Morsani College of Medicine, University of South Florida, Tampa, FL USA

**Keywords:** Malaria, Under-five malaria, Mapping malaria risk, Bayesian methods, Spatio-temporal methods, Integrated Nested Laplace Approximation, INLA, Geospatial methods, Interactive web-based mapping, Sub-Saharan Africa

## Abstract

**Background:**

Ghana is among the top 10 highest malaria burden countries, with about 20,000 children dying annually, 25% of which were under five years. This study aimed to produce interactive web-based disease spatial maps and identify the high-burden malaria districts in Ghana.

**Methods:**

The study used 2016–2021 data extracted from the routine health service nationally representative and comprehensive District Health Information Management System II (DHIMS2) implemented by the Ghana Health Service. Bayesian geospatial modelling and interactive web-based spatial disease mapping methods were employed to quantify spatial variations and clustering in malaria risk across 260 districts. For each district, the study simultaneously mapped the observed malaria counts, district name, standardized incidence rate, and predicted relative risk and their associated standard errors using interactive web-based visualization methods.

**Results:**

A total of 32,659,240 malaria cases were reported among children < 5 years from 2016 to 2021. For every 10% increase in the number of children, malaria risk increased by 0.039 (log-mean 0.95, 95% credible interval = − 13.82–15.73) and for every 10% increase in the number of males, malaria risk decreased by 0.075, albeit not statistically significant (log-mean − 1.82, 95% credible interval = − 16.59–12.95). The study found substantial spatial and temporal differences in malaria risk across the 260 districts. The predicted national relative risk was 1.25 (95% credible interval = 1.23, 1.27). The malaria risk is relatively the same over the entire year. However, a slightly higher relative risk was recorded in 2019 while in 2021, residing in Keta, Abuakwa South, Jomoro, Ahafo Ano South East, Tain, Nanumba North, and Tatale Sanguli districts was associated with the highest malaria risk ranging from a relative risk of 3.00 to 4.83. The district-level spatial patterns of malaria risks changed over time.

**Conclusion:**

This study identified high malaria risk districts in Ghana where urgent and targeted control efforts are required. Noticeable changes were also observed in malaria risk for certain districts over some periods in the study. The findings provide an effective, actionable tool to arm policymakers and programme managers in their efforts to reduce malaria risk and its associated morbidity and mortality in line with the Sustainable Development Goals (SDG) 3.2 for limited public health resource settings, where universal intervention across all districts is practically impossible.

**Supplementary Information:**

The online version contains supplementary material available at 10.1186/s12936-024-04918-x.

## Background

Malaria is a serious global public health problem caused by parasites transmitted to humans via the bites of *Plasmodium*-infected *Anopheles* mosquitoes. According to the World Health Organization (WHO), 558,000 people died from malaria in 2019, among which children under 5 years constituted 74% [1, 2]. Sub-Saharan Africa carried the greatest malaria morbidity and mortality [3], accounting for 95% and 96% of global malaria cases and deaths, respectively, in 2020. Despite intensified malaria control efforts, malaria incidence has risen in recent years, from 229 million in 2019 to 241 million in 2020. Malaria is endemic in Ghana, with notable seasonal variations in its northern ecological zone. The geographical variation in malaria transmission in Ghana is dependent on the duration of the dry season [4]. Data extracted from the District Health Information Management System (DHIMS 2) in Ghana indicated that suspected malaria cases surged from 8 million cases in 2014 to 11 million in 2018 [9]. Likewise, confirmed malaria cases increased from 3.6 million in 2014 to 5.5 million in 2018. Thus, this worsening malaria problem requires tailored and targeted preventive, control, and elimination strategies.

Malaria distribution is highly heterogeneous. Distributing scarce health resources equitably and implementing effective interventions to control malaria requires a deeper understanding of the malaria transmission variations across space and time. Targeted control would benefit significantly from a spatiotemporal statistical model to estimate and map the geographical distribution of malaria over time. Disease risk mapping is a powerful and robust technique for monitoring transmission and control efforts [4]. Likewise, malaria risk mapping is widely applied in spatial epidemiology using geostatistical methods, such as Bayesian hierarchical spatial and spatiotemporal models [5–8]. Previous studies [9–13] in Ghana have applied Bayesian hierarchical spatial and spatiotemporal models to estimate the intensity of malaria transmission across regions of the country. A study mapping the relative risk of malaria in the Greater Accra Region of Ghana from 2015 to 2019 revealed spatial dispersion and seasonal variation with an irregular pattern of malaria transmission across the region [10]. Despite this, none of the studies investigated the spatial distribution of standardized incidence ratio (SIR) of malaria risk across the Ghanaian districts or spatial heterogeneity and clustering of districts with statistically significant high/low malaria risk. Thus, the current study incorporated an interactive web-based spatial disease mapping tool into the Bayesian hierarchical framework to model and map spatiotemporal variation and clustering of relative risk of malaria morbidity in a setting with limited public health resources to arm policymakers and programme managers with an actionable tool for reducing malaria risk and its associated morbidity and mortality in line with the Sustainable Development Goal (SDG) 3.2.

## Methods

### Data source and study population

The current study reviewed and extracted data on uncomplicated confirmed malaria cases and population from the DHIMS2 during 2016–2021. The DHIMS2 is a nationally representative and comprehensive electronic medical record system implemented by the Ghana Health Service (GHS) to collect, collate, report, and analyse routine health service data for the healthcare ecosystem in the country. The DHIMS2 data are aggregated at the district, regional and national levels and comprise both in-patient and out-patient records of suspected and confirmed malaria cases [14]. The required data on uncomplicated confirmed malaria cases in the various health facilities (private, public, and non-governmental) were captured and aggregated at the district level in Ghana. To support the spatiotemporal modelling and mapping, the district shapefile for the 260 districts in Ghana were downloaded from the Database of Global Administrative Areas (GADM) website available at https://gadm.org/download_country.html and terms of use/license available at https://gadm.org/license.html.

### Statistical analysis

The extracted data were transferred to RStudio for data cleaning and analysis. The unit of analysis is district, and there are 260 districts. The population sizes for males and children below 5 years old for 13 districts were not recorded in 2016, 2017, and 2018, and the names of these districts are presented in Additional file 2: Table S1. As a result, multiple imputation (mi) was done to provide values for the missing data points. The Standardized Incidence Rate (SIR) was estimated to assess spatial variation in malaria risk. The districts with SIR values higher than 1 indicated that the risk of malaria morbidity was above what was expected in the standard population, whereas SIR values lower than 1 indicated that the risk of malaria morbidity was lower than what was expected in the standard population. Even though SIR provides important information for determining whether or not the district has a high or low relative risk of malaria, it is not reliable because of errors and white noise in the spatiotemporal data [15]. The samples used in most of the nationally representative sample surveys are not large enough to yield direct and unbiased national estimates for small areas. Furthermore, applying appropriate statistical models can result in greater precision of small area estimates, but this can lead to bias due to misspecified models or ignoring informative sampling [16]. Thus, a sophisticated Bayesian hierarchical spatiotemporal model [17–19], which borrows strength or information across space and time to improve the estimation and prediction of the underlying parameters, was specified to estimate the smooth relative risk of malaria. The Conditional Autoregressive (CAR) and Random Walk of Order one (RW1) were applied to test spatial and temporal correlations between the observations [20–26]. The autocorrelation test was used to determine the presence or absence of spatial and temporal correlations, as the results would justify whether the Bayesian hierarchical spatiotemporal model should be specified. Integrated Nested Laplace Approximation (INLA) was used to estimate the Bayesian hierarchical model [17, 27, 28]. INLA is a powerful estimation method since it combines analytical approximation and numerical integration to obtain the approximated posterior distribution of parameters [18, 29, 30]. The study used uninformative priors on the log-precision of the hyper-parameters because the Bayesian modelling approach required priors, but the study did not have reliable priors for the initial model parameters [23].

The study explored the extent of local clustering of the districts using the local indicator of spatial association (LISA), and mapped statistically significant local clusters to display high-high, low-low, high-low, and low–high clusters. Queen contiguity spatial weights or matrix was used for the LISA statistics [31]. LISA has been described as an important technique for investigating the location of clusters because it helps to estimate and assess statistically significant clusters [32].

### Study variables

The outcome variable was the number of malaria cases aggregated at the district level. The covariate considered in this study included the number of under-five population and the number of male populations in the districts.

### Model description

Let ${Y}_{it}$ denote the number of malaria cases recorded in region $i$ and time period t, ${E}_{it}$ denote the expected number of malaria cases recorded in region $i$ and time period $t$, ${n}_{it}$ denote the number of persons at risk at district $i$ in year $t.$ The expected number of malaria cases recorded in district $i$ and time period $t$ is defined as:

1 $${E}_{it}={n}_{it}\frac{{\sum }_{it}{Y}_{it}}{{\sum }_{it}{n}_{it}}$$

The formula for estimating standardized incidence ratio (SIR) or relative risk (${\theta }_{it}$) of malaria morbidity in each areal unit $i$ at time period $t$ is given as:

2 $${\text{SIR}}= {\widehat{\theta }}_{it}=\frac{{Y}_{it}}{{E}_{it}}$$

The log relative risk is expressed as;

$${\text{log}}\left({\theta }_{it}\right)={\psi }_{it}$$

The Bayesian hierarchical spatiotemporal specification for a Poisson model for counts of malaria cases t ${Y}_{it}$ observed in region $i$ at time period t is defined as:

3 $${Y}_{it}|{e}_{it},{\theta }_{it}\sim Poisson({e}_{it}{\theta }_{it})$$

4 $${{\text{Log}}(\theta }_{it})={\beta }_{0}+{X}_{it}{\prime}\beta +{\mu }_{i}+{\upsilon }_{i}+{\gamma }_{t}+{\varnothing }_{t}$$

$${\beta }_{0}\sim N(0,{\tau }_{0}^{-1})$$

$${\upsilon }_{i}\sim N(0,{\tau }_{\upsilon }^{-1})$$

$${\mu }_{i}|{\left\{{\mu }_{j}\right\}}_{-i}\sim N\left({\overline{\mu }}_{{\delta }_{i}},\frac{{\tau }_{\mu }^{-1}}{{n}_{{\delta }_{i}}}\right),$$

$${\left|{\gamma }_{t}\right|\gamma }_{t-1}\sim N({\gamma }_{t-1}, {\tau }_{\gamma }^{-1})$$

$${\varnothing }_{t}\sim N(0,{\tau }_{\varnothing }^{-1})$$

now ${\tau }_{V}\sim Gamma\left(1, 0.00005\right)$ and ${\tau }_{\mu }\sim Gamma\left(1, 0.00005\right)$, ${\tau }_{\gamma }\sim Gamma\left(1, 001\right)$ and ${\tau }_{\mathrm{\varnothing }}\sim Gamma\left(1, 0.00005\right)$,

where ${\beta }_{0}$ is the overall or average risk for all districts, ${X}_{it}{\prime}$ is a matrix of covariates,$\beta$ denotes unknown corresponding parameter vector coefficients, ${\mu }_{i}$ denotes structured spatial random effect or correlated heterogeneity effect (CH), ${\upsilon }_{i}$ denotes unstructured exchangeable spatial component that models heterogeneity or uncorrelated heterogeneity effect (UH) among the locations at time t. The correlated heterogeneity effect ${\mu }_{i}$ and uncorrelated heterogeneity effect ${\upsilon }_{it}$ are spatial random effects that can vary in time. ${\delta }_{i}$ represents a neighbourhood of the *i*th area, ${n}_{{\delta }_{i}}$ is the number of regions in the *i*th neighbourhood,${\overline{\mu }}_{{\delta }_{i}}$ is the mean of the neighbouring ${\mu }_{i}$ values. ${\gamma }_{t}$ is specified using autoregressive prior distribution and can follow a random walk in time of first order $(\gamma =1),$ which allows for a non-parametric temporal effect. Whereas ${\varnothing }_{t}$ represents an unstructured temporal effect that is independently and identically distributed. All parameters (${\beta }_{0}, {\mu }_{i}, {\upsilon }_{i}, {\gamma }_{t}, {\varnothing }_{t}, {\tau }_{V}, {\tau }_{\mu }, {\tau }_{\gamma }, {\tau }_{\varnothing }$) assigned non-informative prior distribution and their posterior distributions were approximated by INLA. All the analyses were carried out in R-INLA [33].

### Interactive web-based mapping of the predicted malaria prevalence

The study developed an interactive web-based mapping tool to improve the visualization of the predicted malaria risks across the 260 districts and over the 6 years to support the identification of high-risk districts for urgent malaria surveillance and intervention strategies amidst limited public health resources in this setting where universal intervention is practically ineffective and impossible. For each district and year, the study simultaneously mapped the observed malaria counts, district name, standardized incidence rate, and predicted relative risk and their associated standard errors using interactive web-based visualization methods. The *leaflet*, *sp*, and *rgdal* packages in R version 4.2.3 and RStudio were utilized to develop the interactive web-based geospatial maps.

### Ethical consideration

Permission was sought and granted by GHS, who provided the data at no cost for use in this study upon written request.

### The role of the funding source

The present study did not receive any support from any funding source. The authors confirm that they have full access to all the data in this study and accept responsibility for submitting for publication.

## Results

### Malaria trend and transmission in Ghana

The national annual trend of malaria incidence among under-five children in Ghana based on the DHIMS2 data for 2016, 2017, 2018, 2019, 2020, and 2021 are 4,725,597, 5,077,089, 5,774,470, 6,173,507, 5,167,316, and 5,741,261, respectively, resulting in a total malaria case number of 32,659,240 over the six-year period. Notably, malaria cases increased steadily from 2016 to 2019. Although malaria cases decreased at the beginning of COVID-19 in 2020, they increased in 2021 (Table 1).

**Table 1:** National annual trend of malaria incidence among under-five children in Ghana obtained from DHIMS2

Year	Malaria incidence
2016	4,725,597
2017	5,077,089
2018	5,774,470
2019	6,173,507
2020	5,167,316
2021	5,741,261
Total	32,659,240

### Spatiotemporal variation analysis of malaria risk

**Fig. 1:**
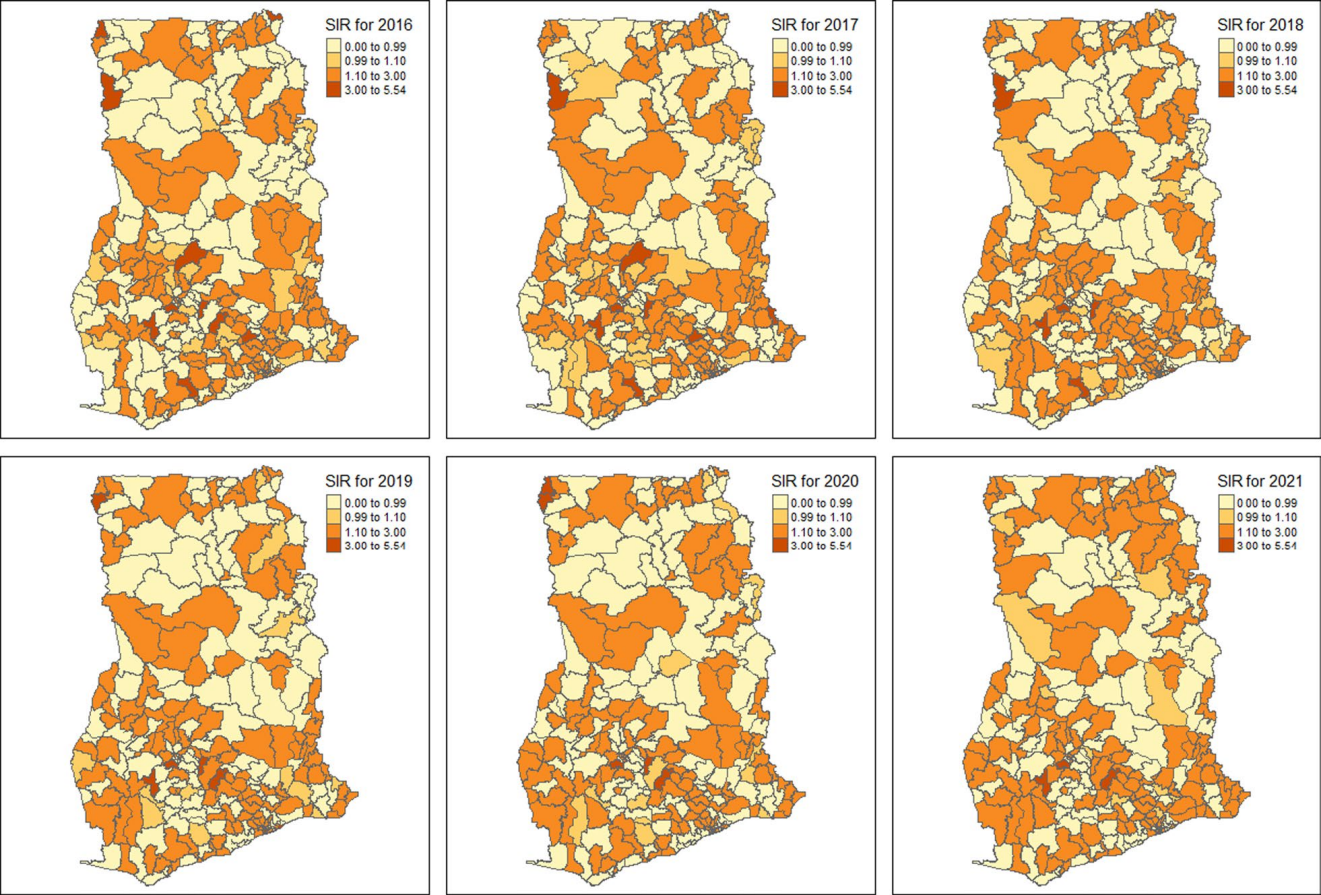
Standardized Incidence Ratio (SIR) of malaria cases across the 260 districts in Ghana (2016–2021)

Figure 1 displays the spatial variation of SIR across the 260 districts in Ghana. The study used the same scale for the SIR of malaria morbidity for the 6 maps for better visualization and comparison. Clusters of districts with a higher risk of malaria morbidity were observed across 2016–2021 in the study. Lawra and Nandom districts in the Upper West region were identified as hotspot areas with consistently high SIR values over the 6 years. Pru East in the Bono East region consistently recorded a high risk of malaria morbidity in 2016–2019 and 2021. Additionally, Anloga, Ketu North, Ketu South districts, and Keta Municipality in the Volta region repeatedly recorded high SIR values for the 6 years. A cluster of districts such as Bole and Central Gonja in the savannah region and Kintampo North Municipality in the Bono East region with high SIR values were observed in 2016–2021.

**Fig. 2:**
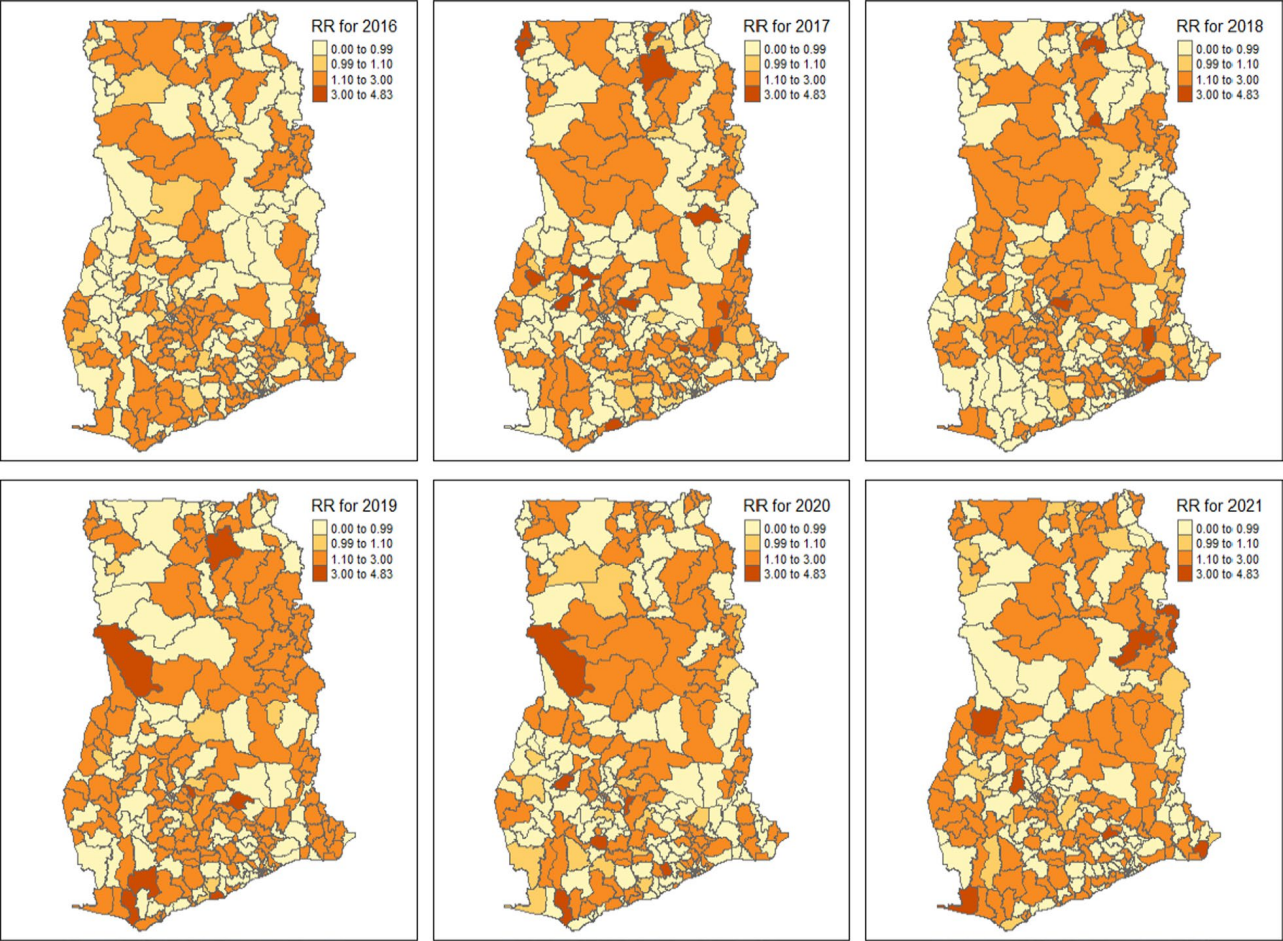
Predicted relative risk of malaria across the 260 districts in Ghana obtained from a weighted Bayesian Hierarchical Spatio-temporal model (2016–2021). (Note: the interactive web-based version of this map is available in Additional file 2 of the online supplementary material)

Figure 2 shows the predicted relative risk of malaria morbidity across the 260 districts in Ghana during 2016–2021. For better visualization, an interactive web-based map version of Fig. 2 is provided in Additional file 1. The average predicted malaria relative risk across all districts from 2016 to 2021 was 1.25 (95% credible interval = 1.23,1.27). The average national relative risk in 2016, 2017, 2018, 2018, 2019, 2020, and 2021 was 1.22 (95% credible interval = 1.20, 1.24), 1.28 (95% credible interval = 1.26,1.29), 1.20 (95% credible interval = 1.18, 1.22), 1.32 (95% credible interval = 1.31, 1.34), 1.20 (95% credible interval = 1.18, 1.21), and 1.27 (95% credible interval = 1.25, 1.29), respectively. The malaria risk is relatively the same over the entire year. However, the highest risk was recorded in 2019 while in the 2021 data, residing in Keta, Abuakwa South, Jomoro, Ahafo Ano South East, Tain, Nanumba North, and Tatali Sanguli districts was associated with the highest risk of malaria burden ranging from a relative risk of 3.00 to 4.83. Spatial clusters of districts with a high or low relative risk of malaria morbidity were observed throughout the six years. The relative risk of malaria in Lawra and Nandom districts in the Upper West region was higher than in the overall population in all the years except for 2020. Additionally, most districts and municipalities in the Savannah, Northern, and Bono East regions recorded a high relative risk of malaria over the entire period. The Bole district in the Savannah region consistently recorded a relative malaria risk above the standard population in 2017, 2018, and 2019. The relative risk of malaria in Kintampo was above average in 2017–2020. Notably, the Bole district was among the lowest malaria risk districts in 2016 (RR: 0.20) but became one of the highest-risk districts in 2019 (RR: 3.74) and 2020 (RR: 3.38) (Table 2).

**Table 2:** Average predicted relative risk of malaria

Year	Posterior mean	95% Credible Interval
2016	1.22	1.20,1.24
2017	1.28	1.26,1.29
2018	1.20	1.18,1.22
2019	1.32	1.31,1.34
2020	1.20	1.18,1.21
2021	1.27	1.25,1.29
Overall	1.25	1.23,1.27

**Fig. 3:**
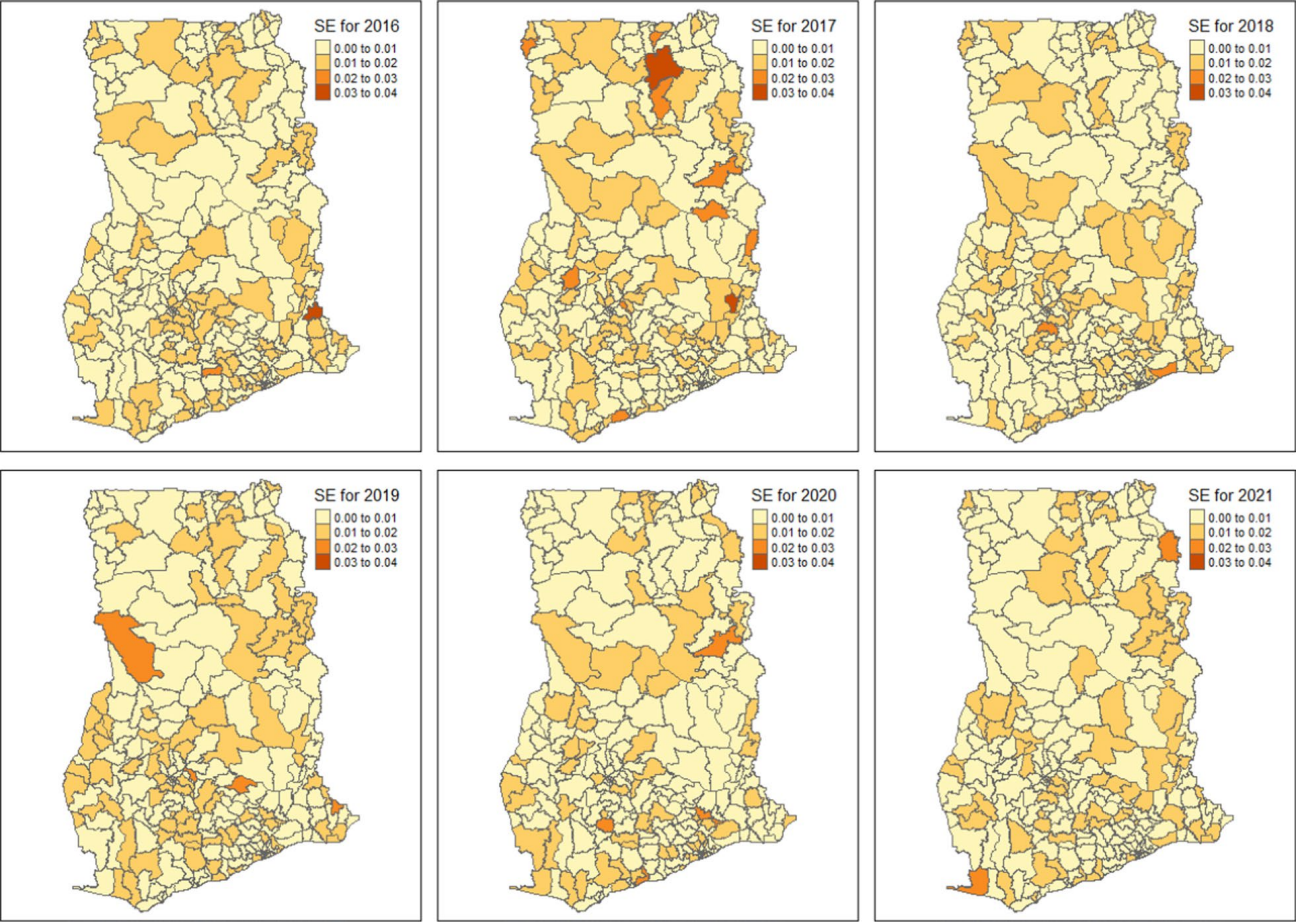
Predicted standard errors associated with the predicted relative risk of malaria across the 260 districts in Ghana obtained from the weighted Bayesian Hierarchical Spatio-temporal model

Presented in Fig. 3 are the predicted uncertainty maps associated with the Smoothed relative risk of malaria across the 260 districts in Ghana obtained from the weighted Bayesian Hierarchical Spatio-temporal model. Generally, the uncertainty associated with the predicted relative risk was very low, ranging from 0.0003 to 0.0323 across the study period. The median and mean standard errors were 0.0081 and 0.0085, respectively.

### Autocorrelation function of malaria cases from 2016 to 2021

**Fig. 4:**
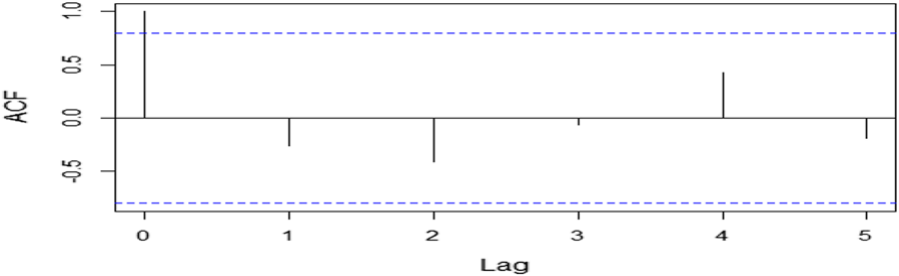
Autocorrelation plot of malaria cases from 2016 to 2021

The autocorrelation plot shows weak autocorrelation of the relative risk of malaria in the districts over the six-year period (Fig. 4). This indicates that malaria cases are not highly correlated.

### The influence of children under 5 and male population on the risk of malaria morbidity

Table 3 shows the covariates associated with the risk of malaria morbidity. Both the log number of children under five years and the log number of males were not significantly associated with altered risks of malaria morbidity. For every 10% increase in the number of children under five years, malaria risk increased by 0.039 (log-mean 0.95, 95% credible interval = − 13.82–15.73) and for every 10% increase in the number of males, malaria risk decreased by 0.075 (log-mean − 1.82, 95% credible interval = − 16.59–12.95). Notably, Bayesian model selection tools such as the deviance information criterion (DIC) and Watanabe–Akaike information criterion (WAIC) scores for Model I and Model II were almost the same even though both model evaluation metrics were in favor of Model I (Table 3).

**Table 3:** Posterior means and Bayesian credible intervals of predicted relative risk of malaria morbidity estimated from the Bayesian Hierarchical Spatio-temporal model with and without covariates (2016–2021)

Parameters	Model 1	Model 2
	Posterior mean (95% Cr.I)	Posterior mean (95% Cr.I)
Fixed effect		
Intercept	− 0.07 (− 0.12, − 0.26)	10.22 (− 4.1 1, 24.55)
Log number of children under 5 years	-	0.95 (− 13.82, 15.73)
Log number of males	–	− 1.82 (− 16.59, 12.95)
Random effect		
${\tau }_{\mu }^{-1}$	20,458.78 (1508.20,6.90×10^+4^)	25,059.51 (1966.54 102,239.83)
${\tau }_{\vartheta }^{-1}$	1.13 (1.05, 1.21)	1.49 (1.39, 1.60)
${\tau }_{\gamma }^{-1}$	127.70 (22.50, 4.25 ×10^+2^)	113.54 (21.32, 318.45)
${\tau }_{\varnothing }^{-1}$	24,762.67 (1251.56, 1.23 ×10^+5^)	27,924.84 (2009.64, 122,507.24
Model evaluation metrics		
DIC	21,118.20	21,121.27
WAIC	20,648.16	20,655.43

Model 1 = Bayesian hierarchical spatio-temporal model without covariates (empty model); Model 2 = Bayesian hierarchical spatio-temporal model with covariates; Cr.I = Credible interval. = Hyperparameter that measures spatial correlation, = Hyperparameter that measures spatial dispersion. ${\tau }_{\gamma }^{-1}$= Hyperparameter that measures temporal correlation, ${\tau }_{\varnothing }^{-1}$ = Hyperparameter that measures temporal dispersion

### Districts with statistically significant high-high, high-low, low–high and low-low values of relative risk of malaria

**Fig. 5:**
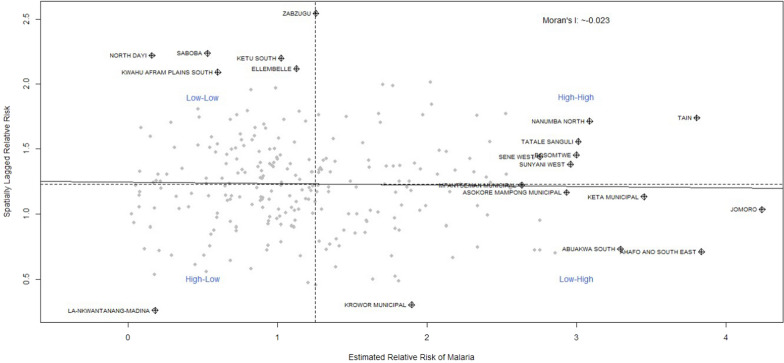
Moran scatterplot of districts with significantly high-high, high-low, low–high and low-low values of relative risk of malaria in 2021

A Moran scatterplot is presented to display clusters of districts with significantly high-high, high-low, low–high, and low-low values of relative risk of malaria from 2016 to 2021. In the upper-right quadrant are districts with elevated levels of risk relative to what would have been expected in the standard population. Additionally, in the lower-left quadrant are districts with significantly lower risk than expected in the standard population (Fig. 5 and Additional file 2: Fig. S1-S5). The Moran scatterplot in Fig. 5 showed that Zabzugu, Nanumba North, Tatale Sanguli, Bosomtwe, Sene West, Sunyani West, and Tain were the districts with high-high risk of malaria in 2021. In Additional file 2: Fig. S1, 7 districts (i.e., Adaklu, Ho West, Agotime Ziope, Ho Municipal, Denkyembour, Bosomtwe, and Efia Kwesimintsim Municipal) in the upper-right quadrant were identified as having statistically significant high relative risk of malaria in 2016. This means that these districts were surrounded by neighbouring districts with high relative risk of malaria. The Additional file 2: Fig. S1 also identified Bunkpurugu Nakpanduri as the only district in the lower-left quadrant with statistically significant low values surrounded by districts with a lower risk of malaria. Additional file 2: Fig. S2 revealed that Jasikan, South Dayi, Afadzato South, Kpando Municipal, and North Dayi districts in the Volta Region recorded statistically significant clustering of high–high relative risk of malaria in 2017. Two districts, including La-Nkwantanang-Madina and Krowor Municipal, fell within the lower-left quadrant with a cluster of low-low risk of malaria in 2017. It is observed in Additional file 2: Fig. S3 that there were 11 districts with statistically significant high-high risk of malaria, while Akatsi North was identified as the only district with low-low risk of malaria in 2018. The results in Additional file 2: Fig. S4 indicated that 10 districts in the upper-right quadrant recorded a significant high relative risk of malaria, surrounded by districts with a high risk of malaria. Clusters of low-low risk of malaria were identified in South Dayi and Agona East in 2019. In 2020, clusters of high-high risk of malaria were recorded in Okere, East Gonja Municipal, Asante Akim North, Yilo Krobo, Kpando Municipal, Bole, and Adansi Akrofuom districts, while a cluster of low-low risk was identified in Dormaa East district (Additional file 2: Fig. S5).

### Spatio-temporal clustering and outliers of the risk of malaria morbidity

**Fig. 6:**
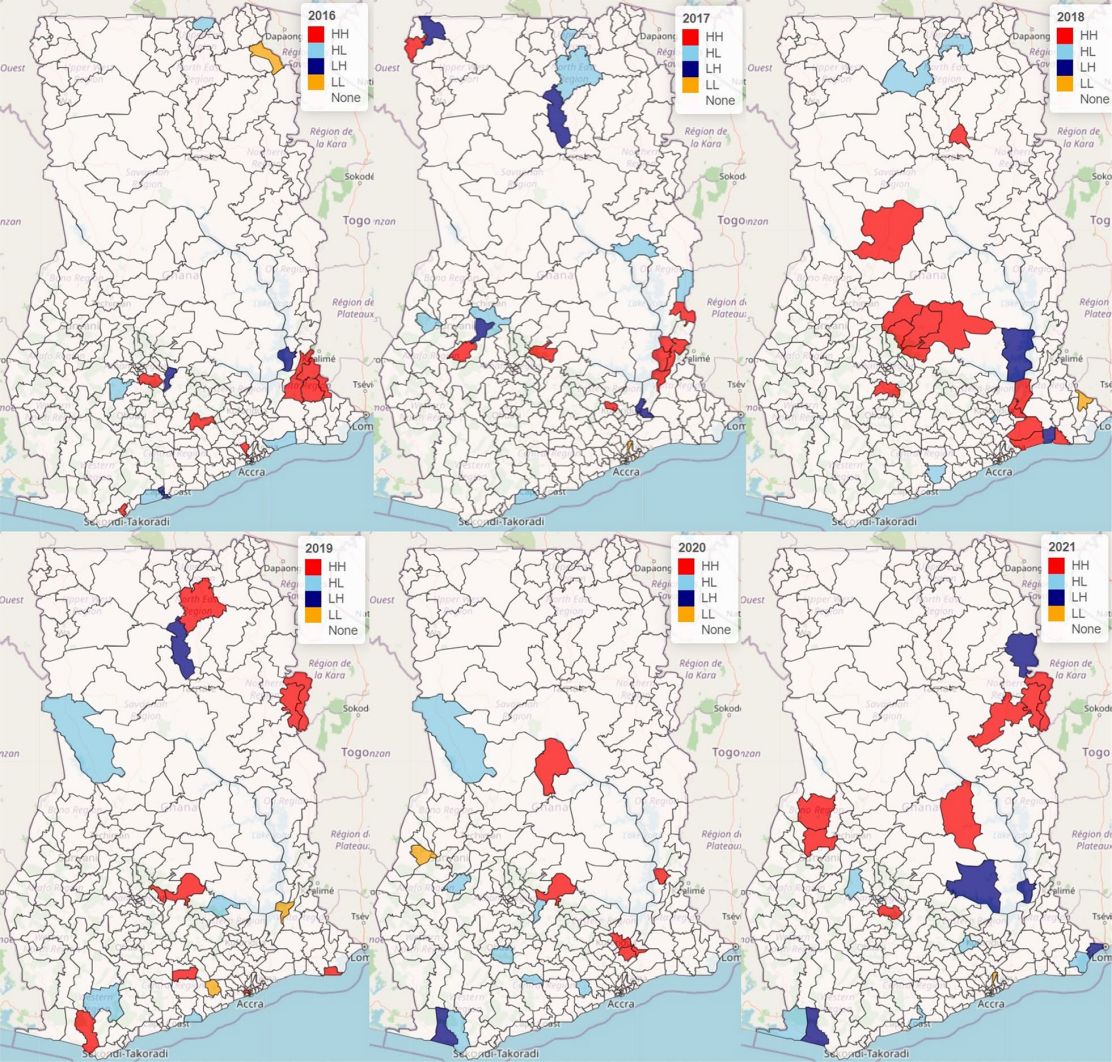
LISA Cluster map showing clusters of districts with high-high (red), high-low (skyblue), low–high (navy), low-low (orange), and significant relative risk of malaria morbidity with associated covariates (2016–2021)

Figure 6 displays the LISA cluster map of districts with extremely high-high, high-low, low–high, and low-low significant relative risks of malaria morbidity with associated covariates from 2016 to 2021. A spatial cluster of significantly high relative risk of malaria morbidity was recorded in the Ho West, Adaklu, and Agotime Ziope districts and Ho Municipality in the Volta region in 2016. The Bosomtwe, Kwaebibirem, Ga North and Effia Kweismintsim, with a significantly high relative risk, shared a high relative risk with neighbouring districts in 2016. The year 2017 identified Lawra, Ahafo Ano North, Sekyere Kumawu, Abuakwa North, Jasikan, Kwahu Afram Plains North, South Dayi, North Dayi, Afadzato South and Kpando Municipal as hot spot districts. In 2018, a significantly high relative risk of malaria morbidity was recorded in the Nanton, Kintampo North Municipal, Ejura-Sekyedumase, Mampong Municipal, Sekyere Central, Sekyere Afram Plains North, Sekyere Kumawu, Bekwai Municipal, Asuogyaman, Lower Manya, Shai Osudoku, Ningo/Prampram and Ada East. In 2019, hotspot districts included West Mamprusi Municipal, Zabzugu, Tatale Sanguli, Asante Akim North, Sekyere East, Achiase, Nzema East, and Anloga, while in 2020, hotspot areas included Kpando, East Gonja, Asante Akim North, Okere and Yilo Krobo districts and municipalities with a significantly high relative risk of malaria morbidity. In 2021, spatial clusters of high-high relative risk of malaria morbidity were observed in Sunyani West and Tain districts in the Bono region, Bosomtwe in the Ashanti region, Sene West districts in the Bono West Region, and Nanumba North, Zabzugu and Tatale Zanguli districts in the Northern Region. It is observed that La Nkwantanang-Madina district, with a significantly low relative risk of malaria morbidity, was surrounded by its neighbours (Ga East, Adenta Municipal and Ayawaso West) with low relative risk. Bosomtwe district in the Ashanti region recorded a statistically significant high relative risk of malaria in 2016 and 2021. Asante Akim North district in the Ashanti region was identified as a hotspot area in 2019 and 2020. A statistically significant high relative risk of malaria was observed in Zabzugu and Tatale Zanguli districts in 2019 and 2021. Sekyere Kumawu district was identified as a hot spot area in 2017 and 2018. South Dayi district recorded a statistically significant high relative risk of malaria in 2017 and this dropped significantly in 2019 (Fig. 6).

## Discussion

The study investigated the district-level geographical and temporal variations in the risk of malaria among children aged < 5 years. The analysis covering the period from 2016 to 2021 revealed three key findings. First, the results showed substantial district-level geographical differences in the risk of malaria across the 260 districts in Ghana. Thus, residing in certain districts was associated with an increased risk of malaria morbidity. Notably, residing in Keta, Abuakwa South, Jomoro, Ahafo Ano South East, Tain, Nanumba North, and Tatale Sanguli districts was associated with the highest burden of malaria risk based on the 2021 data. Secondly, the analysis revealed significant temporal differences in malaria risk by districts in Ghana. Thirdly, significant clustering in spatial patterns of malaria risk was found. Notably, geographical clusters of high-high relative risk of malaria morbidity were found in Sunyani West and Tain districts in the Bono region, Bosomtwe in the Ashanti region, Sene West districts in the Bono West Region, and Nanumba North, Zabzugu and Tatale Zanguli districts in the Northern Region. Geographical and temporal differences and spatial clustering of health outcomes, including malaria, have previously been documented [5–9, 12].

This study developed interactive web-based spatial and temporal malaria risk maps superimposed with observed malaria counts, names of the districts, standardized incidence rates, and relative risks and their associated standard errors. By placing a cursor on any polygon in the interactive web-based maps, the user is automatically provided with these parameters/details about that district for improved visualization and targeted policy and intervention strategies amidst limited public health resources. The color coding alerts the user about the malaria risk levels, with red colors depicting districts with a higher malaria risk. This is a standalone, but effective tool that can be readily available to policymakers and programme managers in charge of malaria prevention, control, surveillance, and elimination efforts in a setting where universal interventions are practically ineffective and impossible due to limited public health resources. To the best knowledge of the authors, this study is the first to have conducted spatiotemporal modelling under INLA supported with interactive web-based spatial mapping of malaria risk in Ghana using routine health service data from 2016 to 2021 covering 260 districts to support efficient and targeted malaria control and elimination efforts in the country.

The average national predicted relative risk across all districts from 2016 to 2021 was 1.25 (95% credible interval = 1.23,1.27), suggesting substantial geographical and temporal differences in malaria risk across the districts in Ghana. The minimum relative risk of 1.20 was recorded in 2018 and 2020, while the maximum of 1.32 was recorded in 2019. The 95% credible interval is relatively wide for some of the random effects, indicating substantial uncertainty which accounts for lack of information. The RR for each year exceeded 1.0, indicating an increased risk of malaria for all the study years. Noticeable changes were also observed in the risk of malaria for some districts over some periods in the study. For example, the Bole district was among the lowest-risk districts in 2016 (RR: 0.20), but one of the highest-risk districts in 2019 (RR: 3.74) and 2020 (RR: 3.38). On the other hand, West Mamprusi had the highest RR in 2017 (RR: 4.43) and 2019 (RR: 3.08), but the lowest RR in 2020 (RR: 0.96). These findings support previous studies that examined spatial and temporal variations and found substantial spatial and temporal differences in malaria morbidity [5, 7, 9–12]. The finding that some districts are at the highest risk of malaria morbidity indicate serious threats to the progress in malaria control in Ghana, and interventions are urgently required to address this. It is imperative to note that the DIC and WAIC scores are similar for Bayesian hierarchical spatio-temporal model without covariates (model 1) and Bayesian hierarchical spatio-temporal model with covariates (model 2). This might be due to the fact that the two covariates used in model 2 did not account for extra variability in the relative risk of malaria.

The use of routine health service data, which are more frequently collected, provides high-quality data for more timely investigation of health outcomes and disease burdens like malaria to inform timely interventions based on new evidence, unlike surveys that are conducted every five years like the Demographic and Health Surveys and the Multiple Indicator Cluster Surveys.

The modelling and mapping approach is a critical and relevant tool to guide policymakers and programme managers in the development of targeted policies and intervention strategies that can help improve malaria risk surveillance to ensure that the implementation of policies, interventions, and programmes are targeted at districts with the utmost need and at the right time. This tool uses routine health service data to effectively monitor changes in malaria transmission which can be used to evaluate progress.

## Conclusion

This study provided a critical tool for planning the optimal and efficient allocation of scarce resources, surveillance, and evaluation of malaria control policies, interventions and programmes. The approach used in this study permits web-based maps to be created and provides key information on the contribution of geographical location and time on malaria burden across the 260 districts in Ghana. This approach can be applied to other substantial public health challenges such as HIV/AIDS, TB, malnutrition, and vaccination, in Ghana and other settings. The higher burden malaria risk districts identified should be urgently targeted with additional but effective malaria control programmes to reduce the risk of malaria morbidity and its associated mortality. The use of spatiotemporal modelling and interactive web-based mapping of malaria risk using routinely collected health service data to examine goals/targets set for malaria risk reduction at the district level is highly recommended. Further studies are warranted to search for additional factors not considered in this study that might explain why some districts were at higher risk of malaria morbidity while others are not, as part of the overall strategy in addressing the problem of under-five malaria morbidity and its associated mortality.

## Limitations of the study

This study could not consider all potential district-level predictors of malaria risk in the study because the DHIMS2 database had limited variables that could be included in the model to explain some of the increased relative risks, For example. environmental and geographical factors were not considered because the DHIMS2 which is the source of the required data used does not capture these types of variables. The two covariates were the only covariate captured in the DHIMS. This is one of the limitations of the current study.

### Supplementary Information

**Additional file 1.** Figures for supplementary material for online interactive web-based map for Fig. 2 presented in the manuscript.

**Additional file 2.** Additional Table S1 and Figures S1–S5.

## Data Availability

The datasets generated and/or analysed during the current study are provided freely by the Ghana Health Service and are available upon official written request to the Director General of the Ghana Health Service. The contact details are available at the Ghana Health Service website https://ghs.gov.gh/.

## Abbreviations

DHIMS2: District Health Information Management System II
DIC: Deviance Information Criterion
GHS: Ghana Health Service
INLA: Integrated Nested Laplace Approximation
SSA: Sub-Saharan Africa
SDG: Sustainable Development Goals
WAIC: Watanabe-Akaike information criterion
