# Supplementary material for: Modelling spatiotemporal variation in under-five malaria risk in Ghana in 2016–2021
Source: Malar J. 2024 Apr 9;23:102. doi: 10.1186/s12936-024-04918-x (PMC11005246; doi:10.1186/s12936-024-04918-x)
Supplement: Supplementary file 2 — Additional file 2. Additional Table S1 and Figures S1–S5. [file 12936_2024_4918_MOESM2_ESM.docx]

Table 1 Districts with missing population sizes for males and children <5 years for 2016, 2017 and 2018

| Number | Districts |
| --- | --- |
| 1 | Ablekuma Central |
| 2 | Ablekuma North |
| 3 | Ablekuma West |
| 4 | Asokwa |
| 5 | Ayawaso East |
| 6 | Ayawaso North |
| 7 | and Tema West |
| 8 | Ayawaso West |
| 9 | Kwadaso |
| 10 | Oforikrom |
| 11 | Okaikwei North |
| 12 | Old Tafo |
| 13 | Suame |


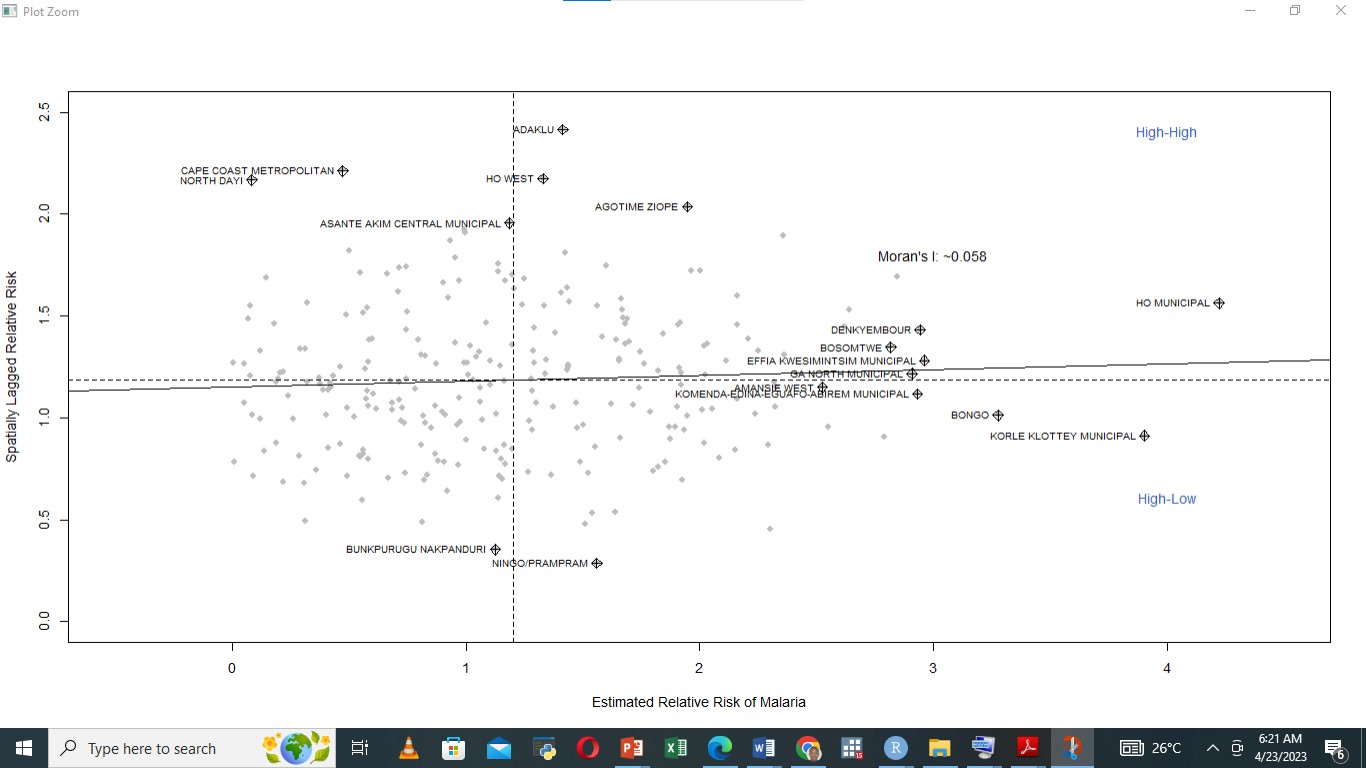


Fig. 1 Moran scatterplot of districts with significantly high-high, high-low, low-high and low-low values of relative risk of malaria in 2016


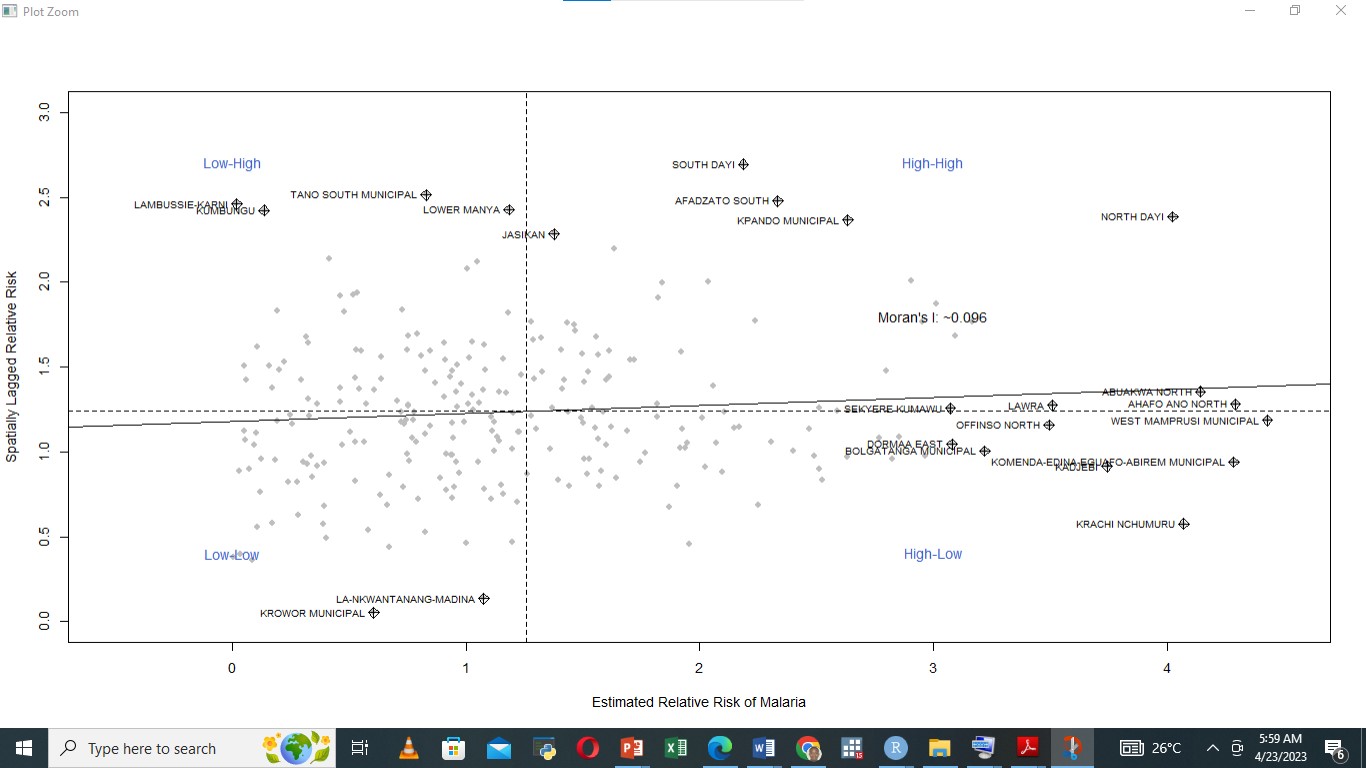


Fig. 2 Moran scatterplot of districts with significantly high-high, high-low, low-high and low-low values of relative risk of malaria in 2017


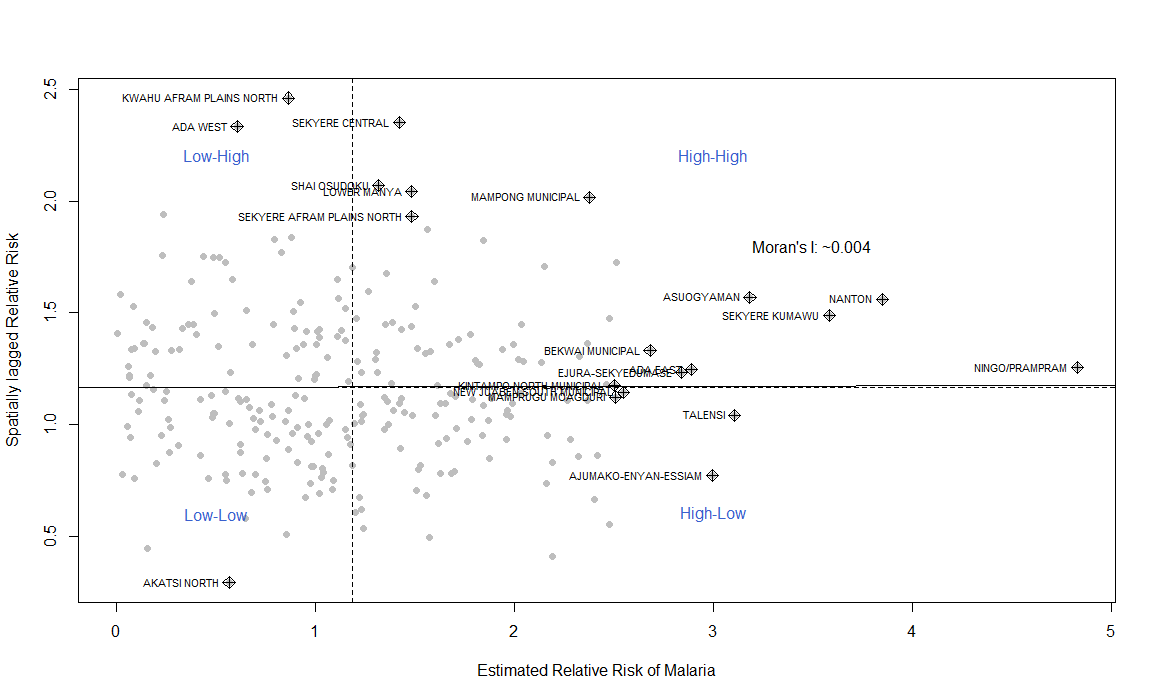


Fig. 3 Moran scatterplot of districts with significantly high-high, high-low, low-high and low-low values of relative risk of malaria in 2018


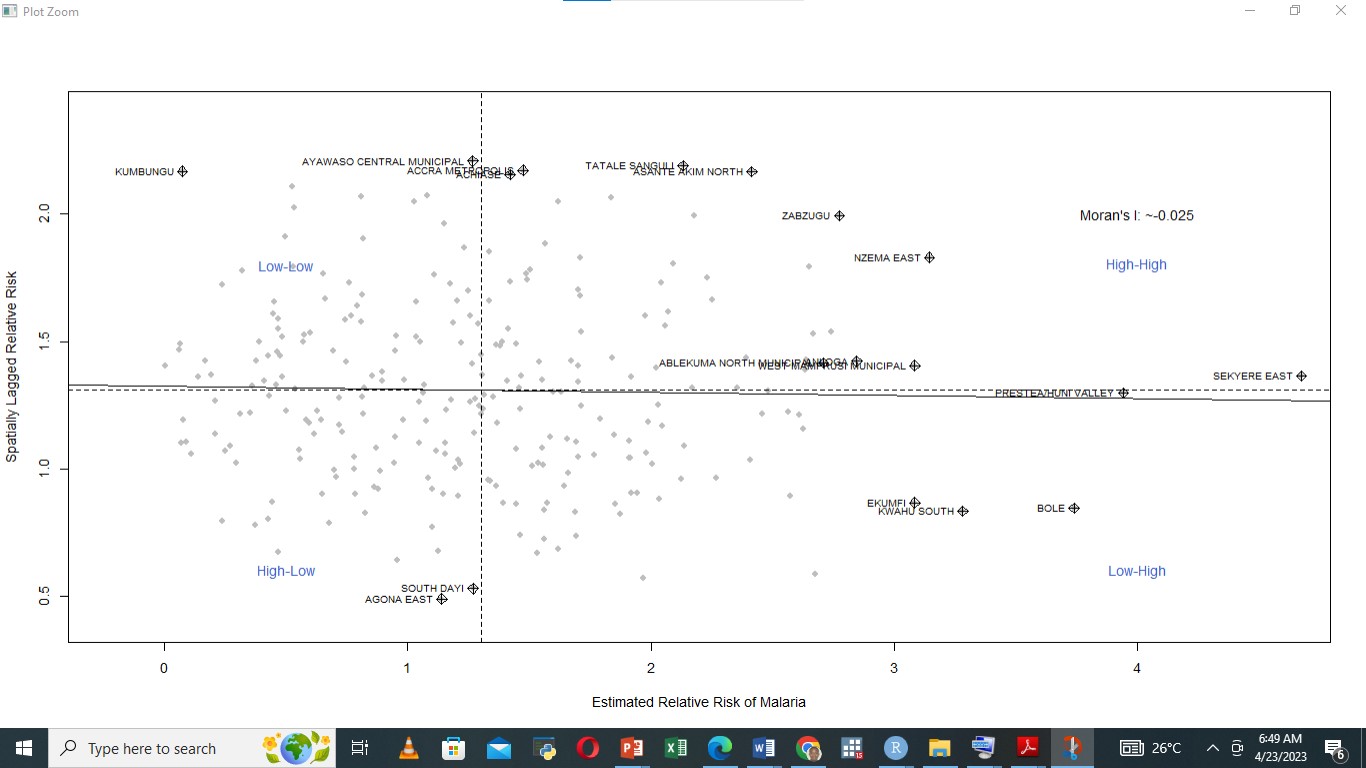


Fig. 4 Moran scatterplot of districts with significantly high-high, high-low, low-high and low-low values of relative risk of malaria in 2019


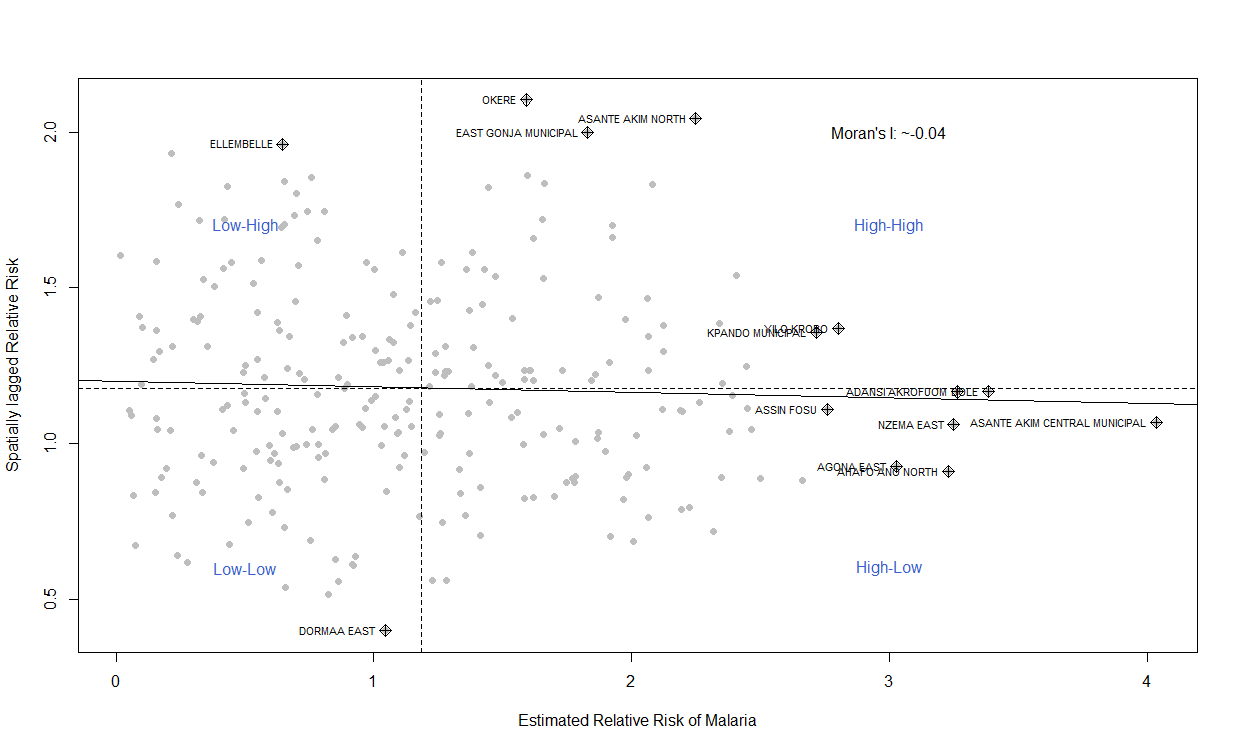


Fig. 5 Moran scatterplot of districts with significantly high-high, high-low, low-high and low-low values of relative risk of malaria in 2020
